# Drought stress modify cuticle of tender tea leaf and mature leaf for transpiration barrier enhancement through common and distinct modes

**DOI:** 10.1038/s41598-020-63683-4

**Published:** 2020-04-21

**Authors:** Mingjie Chen, Xiaofang Zhu, Yi Zhang, Zhenghua Du, Xiaobing Chen, Xiangrui Kong, Weijiang Sun, Changsong Chen

**Affiliations:** 1 Henan Key Laboratory of Tea Plant Biology, College of Life Sciences, Xinyang Normal University, Xinyang, Henan 464000 China; 2 Tea Research Institute, Fujian Academy of Agricultural Sciences, Fuan, Fujian 355000 China; 3 Horticultural Plant Biology and Metabolomics Center, Haixia Institute of Science and Technology, Fujian Agriculture and Forestry University, Fuzhou, Fujian 350002 China; 4 Anxi College of Tea Science, Fujian Agriculture and Forestry University, Fuzhou, Fujian 350002 China

**Keywords:** Waxes, Drought

## Abstract

Cuticle is the major transpiration barrier that restricts non-stomatal water loss and is closely associated with plant drought tolerance. Although multiple efforts have been made, it remains controversial what factors shape up the cuticular transpiration barrier. Previously, we found that the cuticle from the tender tea leaf was mainly constituted by very-long-chain-fatty-acids and their derivatives while alicyclic compounds dominate the mature tea leaf cuticle. The presence of two contrasting cuticle within same branch offered a unique system to investigate this question. In this study, tea seedlings were subjected to water deprivation treatment, cuticle structures and wax compositions from the tender leaf and the mature leaf were extensively measured and compared. We found that cuticle wax coverage, thickness, and osmiophilicity were commonly increased from both leaves. New waxes species were specifically induced by drought; the composition of existing waxes was remodeled; the chain length distributions of alkanes, esters, glycols, and terpenoids were altered in complex manners. Drought treatment significantly reduced leaf water loss rates. Wax biosynthesis-related gene expression analysis revealed dynamic expression patterns dependent on leaf maturity and the severity of drought. These data suggested that drought stress-induced structural and compositional cuticular modifications improve cuticle water barrier property. In addition, we demonstrated that cuticle from the tender leaf and the mature leaf were modified through both common and distinct modes.

## Introduction

The cuticle presents on the outer surface of the epidermal cells at the aerial surfaces of vascular plants except the stems of woody plants, it is constituted of cutin and waxes which collectively form a hydrophobic layer. Cutin is insoluble polyester of long-chain hydroxyl fatty acids; waxes are either embedded within the cutin matrix in the form of intracuticular waxes or deposited on the outer surface as an epicuticular film, and are soluble in organic solvents^1,2^. Cuticular waxes vary qualitatively and quantitatively among plant species; within same species wax composition also is organ-, tissue-, or even developmental stage- dependent^3–8^. Based on cuticular wax composition plants can be broadly divided into two groups: plants containing only very long chain fatty acids (VLCFAs) and their derivatives such as alcohols, alkyl esters, aldehydes, and alkanes in their cuticular waxes, and plants with high percentage of alicyclic compounds (triterpenoids, steroids, or tocopherols) besides VLCFAs^9^. Recently, Zhu *et al*.^3^ reported that cuticular waxes from tender tea leaf mainly contain VLCFAs without triterpenoids; in contrast, cuticular waxes from mature tea leaf are dominated by triterpenoids and steroids.

Plant cuticle plays multiple functions in the interactions with environment, its principal function is to restrict uncontrolled water loss through non-stomatal pathway^10,11^. Studies from diverse plant species have demonstrated that the cuticle thickness and overall waxes load do not positively correlate with its transpiration barrier^9,12,13^. However, total resistance was reported to correlate with the percentage of aliphatic compounds within the intracuticular wax mixtures, and intracuticular resistance was negatively associated with alicyclic compounds in the intracuticular waxes^9^. Studies from artificial membrane also demonstrated that alkanes, alcohols, and aldehydes conferred greater resistance to water diffusion than either VLCFAs or the triterpenoids oleanolic and ursolic acid^14^.

Tea tree (*Camellia sinensis* [L.] O. Kuntze) is perennial evergreen woody crop species with life span over 100 years, its leaves can last around one year after bud break^15^. Tea tree is commonly grown in rain-shed ecosystems, and inevitably encounters seasonal drought. In fact, drought is one of the major environmental factors to constrain tea growth. Previous studies from diverse plant species have demonstrated that drought-induced cuticle modifications are species- or genotype- specific^16–23^. By now, most researches on tea drought stress response are concentrated on the morphological, physiological, biochemical, or molecular mechanisms^24–26^, few studies investigate its cuticle contributions to drought tolerance. Interestingly, tender tea leaves showed very different cuticular wax composition compared to fully expanded mature leaves: triterpenoids are abundantly present in cuticular waxes of mature leaf but absent from that of tender leaf^3^. This characteristic wax distribution pattern offered a unique system to dissect the relationship between wax lipid composition and cuticular transpiration barrier properties. We hypothesized that by observing how the cuticle of tender leaf and mature leaf respond to drought stress, one could uncover the factors that contribute to the cuticular transpiration barrier. In this article, one-year-old tea trees (*Camellia sinensis cv Jinmudan*) were subjected to drought treatment by withholding water for 15 days, cuticles from the tender second leaf and the mature fifth leaf were compared at morphological, biochemical, and molecular levels during water-deprivation treatment. We found that besides common cuticular modifications shared by both types of leaves, leaf maturity-specific cuticle changes also were uncovered. Our data demonstrated that the tender leaf and the mature leaf adopted some common and specific cuticular wax modifications for the enhancement of transpiration barrier under drought stress.

## Results

### Tea tree morphological changes under drought stress

**Figure 1:**
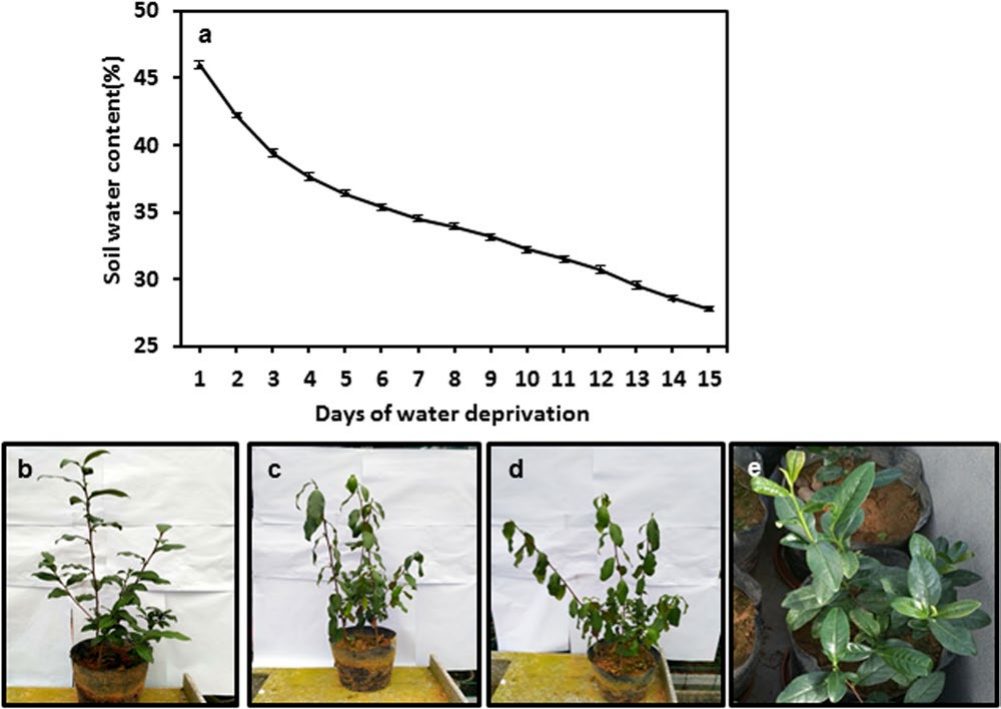
Changes in soil water contents during water deprivation treatment and morphological changes of tea tree. (**a**) Pot soil water content changes. (**b**–**d**) Tea tree performance at the first day, the eighth day, and the fifteenth day after the initiation of water deprivation treatment, respectively. (**e**) The well-watered control plants at D-15.

The optimum relative soil water contents for *Camellia sinensis* were in the ranges of 70–90% (Yang, 2005), this is equivalent to absolute soil water contents of 32%-42% of red soil. Before the initiation of water withholding the tea pots were fully irrigated to ensure even soil water content for all pots. At the first day of water withholding (D-1) the absolute soil water content was 46%. Within the first three day of water withholding, the soil water content dropped at faster rate, then decreased at relatively constant rate (Fig. 1a). At D-8, the soil water content reached to 34%, the lower threshold for optimal growth, tea tree started to show symptoms of water shortage: the leaves started to droop at noon (Fig. 1c), then the turgor pressure recovered throughout the night period. At D-15, the soil water content dropped to 28%, the wither symptoms were further exacerbated: the leaves started to droop earlier in the morning, and the turgor pressure recovered slower during night period; stem apical buds became dormant, no new leaves emerged; the leaf color turned into pale yellow, mature leaves at lower part of the plants started to senesce (Fig. 1d). In contrast, well-watered control plants grew normally (Fig. 1e). These observations suggested that the tender leaf was more tolerant to drought stress compared to the mature leaves.

### The epicuticular wax crystals from the tender leaf and the mature leaf were differentially affected by drought stress

**Figure 2:**
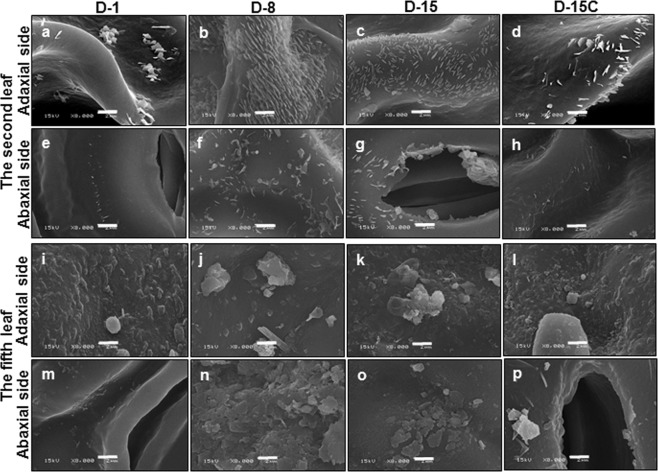
The scanning electron microscopy of the second leaf and the fifth leaf during water deprivation treatment. D-1, D-8, and D-15 represent the first day, the eighth day, and the fifteenth day of water deprivation, respectively; D-15C: the well-watered control plants at day 15. Bar = 2 µm.

After bud break new leaves are sequentially emerged, thus form a developmental gradient along the new branch: the leaves next to the apical bud are newly developed and are more tender; in contrast, the leaves located at the lower part of the branch are emerged earlier thus more mature. Along this developmental gradient, Zhu *et al*.^3^ demonstrated that leaf cuticular wax experienced dramatic compositional changes with leaf maturation: alicyclic compounds did not present from the tender second leaf but dominate the mature fifth leaf. Thus, in this study only the second leaf and the fifth leaf were selected to represent the tender leaf and mature leaf, respectively. At D-1, the epicuticular wax crystals from the tender second leaf only sparsely distributed across the adaxial and abaxial surfaces (Fig. 2a,e); at D-8, the adaxial surfaces were densely covered with 1–2 µm rod-like wax crystals (Fig. 2b). The wax crystal density at the abaxial surface also was increased compared with D-1 (Fig. 2e,f), but at much lower level compared with the adaxial surface (Fig. 2b,f). The crystals on the adaxial surface showed distribution patterns likely representing cuticular ridges^18^ (Fig. 2b). In contrast, the wax crystals were randomly distributed on the abaxial surface. From D-8 to D-15, epicuticular wax crystals were not increased further (Fig. 2c,g). At D-15, wax crystals were observed from guard cells. In contrast, the epicuticular surfaces of the well-watered control plants did not show significant difference with the plants at D-1 (Fig. 2d,h), suggesting that these changes in epicuticular wax crystals were resulted from water deprivation treatment.

For the fully expanded fifth leaf, at D-1both leaf surfaces were covered with papillae-shaped crystals, a higher crystal density was observed from the adaxial surface compared with the abaxial surface (Fig. 2i,m). At D-8, the wax crystal density from adaxial surface was not increased, but crystal size became larger (Fig. 2j); in contrast, plates-like crystals were appeared on abaxial surface (Fig. 2n). At D-15, wax crystal density from both surfaces was not increased further compared with D-8 (Fig. 2k,o).

### Cuticle ultrastructure and thickness from the tender leaf and the mature leaf were altered by drought stress

**Figure 3:**
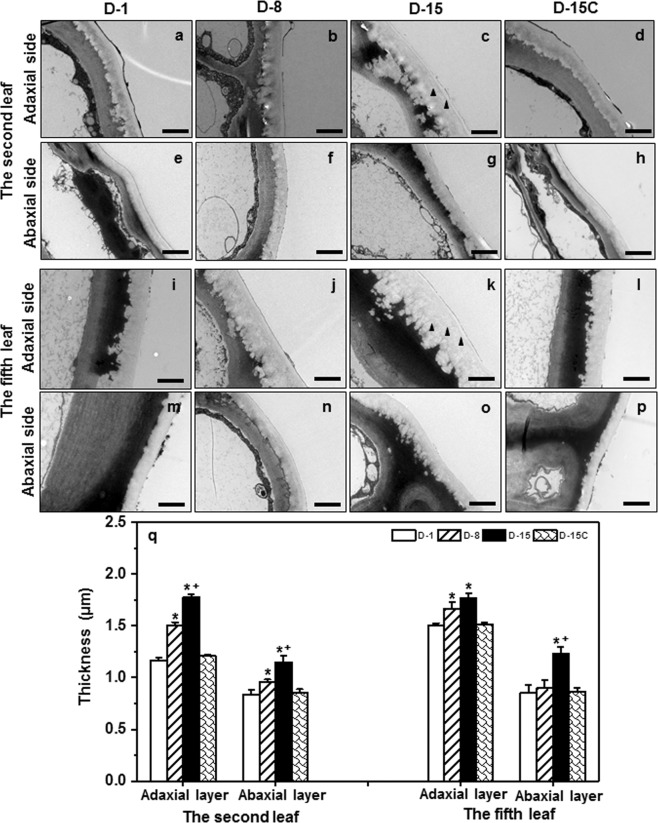
The changes of cuticle thickness and ultrastructure from the second and the fifth leaf during water deprivation treatment. Black arrows indicate electron-dense lamellae structure. D-1, D-8, and D-15 represent the first day, the eighth day, and the fifteenth day of water deprivation treatment, respectively; D-15C: the well-watered control plants at day 15. *Indicates D-8 or D-15 was statistically significant compared with D-1 (p < 0.05); ^+^ indicates D-15 was statistically significant compared with D-8 (p < 0.05).

Water deprivation-induced cuticle ultrastructural changes were observed under transmission electron microscopy (TEM). Cuticular ridges and groves were observed between the interface of cell wall and cuticle, and their size increased from D-1 to D-15 (Fig. 3a–c,i–k,m–o). Generally, these structures were more evident from the adaxial surface (Fig. 3ck) compared to the abaxial side (Fig. 3g,o). At D-15, electron-dense lamellae structures within cuticle became more visible especially from the adaxial surface (Fig. 3c,k, indicated by solid arrow). These electron dense layers were proposed to be cutin proper^27^. This may suggest that cutin content could be increased with the progression of water deprivation.

Cuticle thickness was measured from TEM images, the data clearly demonstrated that cuticle thickness was increased with the progression of water deprivation (Fig. 3q). The tender leaf showed faster increase on cuticle thickness compared to the mature leaf. At D-1, the adaxial cuticle thickness from the second leaf was thinner than that of the fifth leaf. At D-8 and D-15, its thickness increased 29% and 52%, respectively. In contrast, the adaxial cuticle thickness from the fifth leaf increased only 11% and 18%, respectively. As a result, at D-15 there was no significant difference between the adaxial cuticle thickness from the second leaf and the fifth leaf. At D-15, the cuticle thickness from the well-watered control plants did not show significant difference with plants at D-1 (Fig. 3q).

The abaxial cuticle from the second leaf showed steady increase from D-1 to D-15; in contrast, the abaxial cuticle from the fifth leaf did not show significant changes during the first 8-day of water deprivation. At D-1, the fifth leaf abaxial cuticle was slightly thicker than that of the second leaf; however, at D-8 it became thinner than that of the second leaf. These observations suggested that the tender leaf was more sensitive to soil water content decline compared to mature leaf.

### Cuticular wax coverage from the tender leaf and the mature leaf increased by drought stress

**Figure 4:**
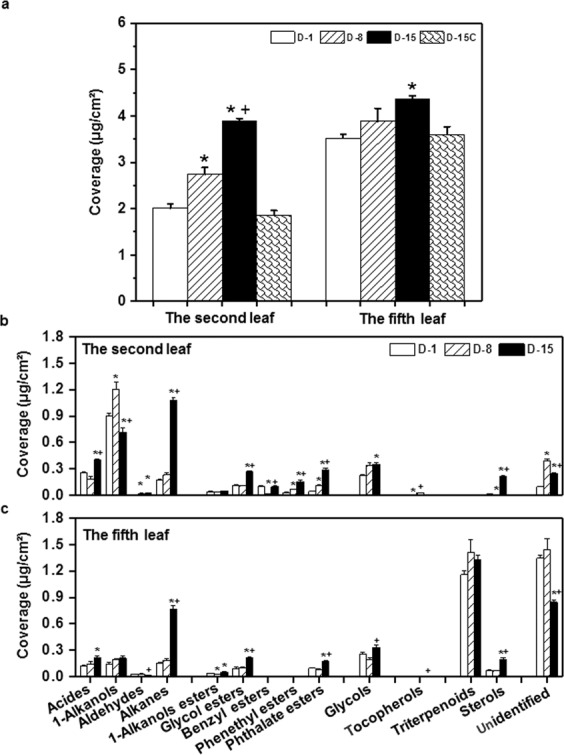
The wax coverage changes of the second leaf and the fifth leaf during water deprivation treatment. D-1, D-8, and D-15 represent the first day, the eighth day, and the fifteenth day of water deprivation, respectively; D-15C: the well-watered control plants at day 15. *Indicates D-8 or D-15 was statistically significant compared with D-1 (p < 0.05); ^+^ indicates D-15 was statistically significant compared with D-8 (p < 0.05).

Total waxes coverage of the tender leaf increased from 2.01 µg cm^−2^ to 3.89 µg cm^−2^ during the 15-day of water deprivation, which account for a total 93.5% increase. In contrast, within same period the waxes coverage from the fifth leaf was increased only 24% (Fig. 4a). The waxes coverage from the well-watered control plants was not altered at D-15 compared to that of D-1 (Fig. 4a).

Tea leaf cuticular waxes contained seven chemical classes, included acids, 1-alkanols, aldehydes, alkanes, esters, glycols, and terpenoids^3^. Wax compositions were monitored during water deprivation treatment. From D-1 to D-8, alkanes, acids, 1-alkanol esters, glycol esters, and glycols showed little changes from both leaves. However, from D-8 to D-15 these wax components were dramatically increased (Fig. 4b,c). The second leaf and the fifth leaf showed some distinct compositional changes of cuticular waxes. The 1-alkanols content from the tender leaf was first increased at D-8 followed by a decrease at D-15; accordingly, the acids were slightly decreased at D-8 followed by an increase at D-15 (Fig. 4b). This changing pattern likely suggested that in the tender leaf the VLCFA reduction pathway, which converts acids into 1-alkanols, was activated during the first 8-day of water deprivation, then was deactivated. In contrast, 1-alkanol contents from the fifth leaf kept unchanged during water deprivation (Fig. 4c). The alkanes and esters from both leaf positions showed rapid increase from D-8 to D-15, suggesting that most acids were diverted into fatty acid decarboxylation pathway or ester formation pathway in response to water deficient. The triterpenoids from the fifth leaf did not show significant changes during water deprivation. However, the steroid contents from both leaves were significantly increased from D-8 to D-15 (Fig. 4a,b).

### Wax chain length distribution was altered by drought stress

**Figure 5:**
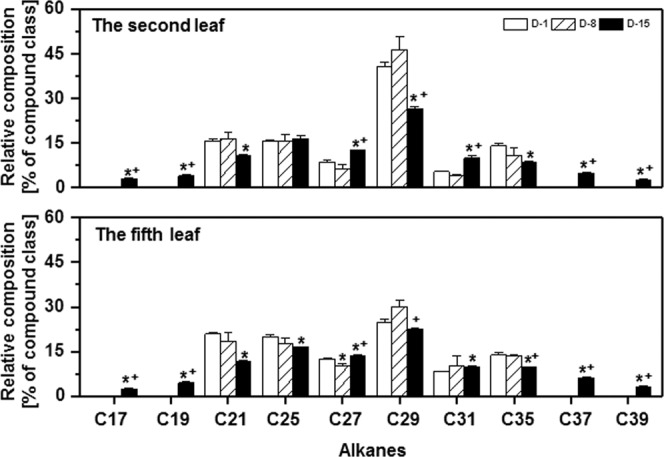
Chain length distributions of alkanes during water deprivation treatment. D-1, D-8, and D-15 represent the first day, the eighth day, and the fifteenth day of water deprivation, respectively; D-15C: the well-watered control plants at day 15. *Indicates D-8 or D-15 was statistically significant compared with D-1 (p < 0.05); ^+^ indicates D-15 was statistically significant compared with D-8 (p < 0.05).

As we showed above, the absolute contents of alkanes, esters, glycols, and steroids were significantly increased by water deprivation treatment, their chain length distributions were examined further. Under normal growth conditions, alkane carbon chain length include C_21_, C_25_, C_27_, C_29_, C_31,_ and C_35_. At D-8, alkane composition was not significantly affected from both leaf positions. However, at D-15, chain length was differentially altered, with C_21_, C_29,_ and C_35_ decreased, meanwhile C_27_ and C_31_ increased from both leaf positions (Fig. 5). Interestingly, at D-15 shorter chain lengths (C_17_ and C_19_) as well as longer chain lengths (C_37_ and C_39_) were detected from both leaf positions (Fig. 5).

The ester fraction includes 1-alkanol esters, glycol esters, benzyl esters, phenethyl esters, and phthalate esters (Fig. S1a)^3^. In the tender leaf, C_29_ benzyl esters at D-8 decreased significantly compared with D-1, meanwhile phenethyl esters and C_24_ phthalate esters increased. At D-15, the percentage of C_22_ 1-alkanol ester, C_18_ and C_21_ glycol esters was significantly decreased, while C_19_ glycol ester increased (Fig. S1a, upper panel). In the mature leaf, only 3 esters were detected, they are: 1-alkanol esters, glycol esters, and phthalate esters. At D-8, no esters were significantly affected; at D-15, the fractions of C_19_ glycol ester increased significantly, while C_21_ glycol esters and C_16_ phthalate esters decreased (Fig. S1a, lower panel).

Under normal growth condition, C_16_, C_18_, C_20,_ and C_22_ glycols were commonly detected. The tender leaf and the mature leaf showed similar changes in C_16,_ C_18,_ and C_22_ glycols. C_16_ glycol was decreased with the progression of water deprivation; C_18_ glycol kept unchanged at D-8, then increased at D-15, while C_22_ glycol was increased at D-8 followed by a decrease at D-15. C_20_ glycol fraction was not affected by water deprivation (Fig. S1b, upper panel); however, in the mature leaf C_20_ glycol was increased at D-8 followed by a decrease at D-15 (Fig. S1b, lower panel). Interestingly, C_24_ glycol was detected from both leaf positions only at D-15 with a concurrent decrease of C_22_ glycol. These data suggested that C_22_ glycol could be the precursor for C_24_ glycol biosynthesis (Fig. S1b).

Four different steroids were detected from the wax mixtures of the mature leaf, including campesterol, stigmasterol, lanosterol, and 24-methylenecycloartanol, while only stigmasterol was detected from the tender leaf (Fig. S2). At D-15, β-sitosterol was detected from both leaves but undetectable at D-1 or D-8 (Fig. S2). In both leaves stigmasterol coverage first decreased at D-8 followed by an increase at D-15 (Table S1). However, its percentage was significantly reduced during water deprivation, mainly due to the dramatic increase of β-sitosterol at D-15 (Fig. S2). In the mature leaf, lanosterol first increased at D-8 followed by a decrease at D-15; 24-methylenecycloartanol was significantly increased at D-15 (Fig. S2; Table S1).

Triterpenoids were detected from the mature leaf and absent from the tender leaf (Fig. S2). Under regular growth condition, triterpenoids include α-amyrin, β-amyrin, lupeol, ursolic acid, friedelin, and betulin (Fig. S2 bottom panel). At D-8, β-amyrin dramatically decreased by 96%, while betulin increased 36.6 folds (Table S1). At D-15, majority of the triterpenoids were lower than that of D-1. Interestingly, two new triterpenoids (β-amurone and canophyllol) were detected at D-8 and D-15, respectively (Fig. S2). β-amurone and canophyllol have been reported from tea previously^3,28^.

### Leaf water loss was reduced by drought treatment

**Figure 6:**
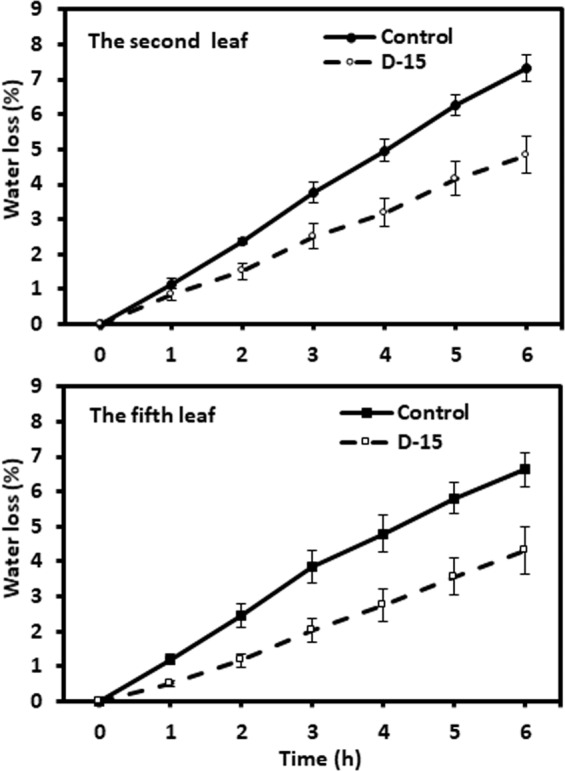
Water loss rates of the second leaf and the fifth leaf from well-watered control plants and drought treated plants at day 15.

To observe if water deprivation-induced cuticle modifications affect its transpiration barrier properties, the water loss rates were measured from the tender leaf and the mature leaf at D-15. We found that the leaf water loss rates from drought treated leaves were significantly decreased compared with that of the well-watered control leaves (Fig. 6). These data suggested that the drought-induced cuticle modifications improved cuticle barrier properties.

### The expression of wax genes was differentially affected by drought

**Figure 7:**
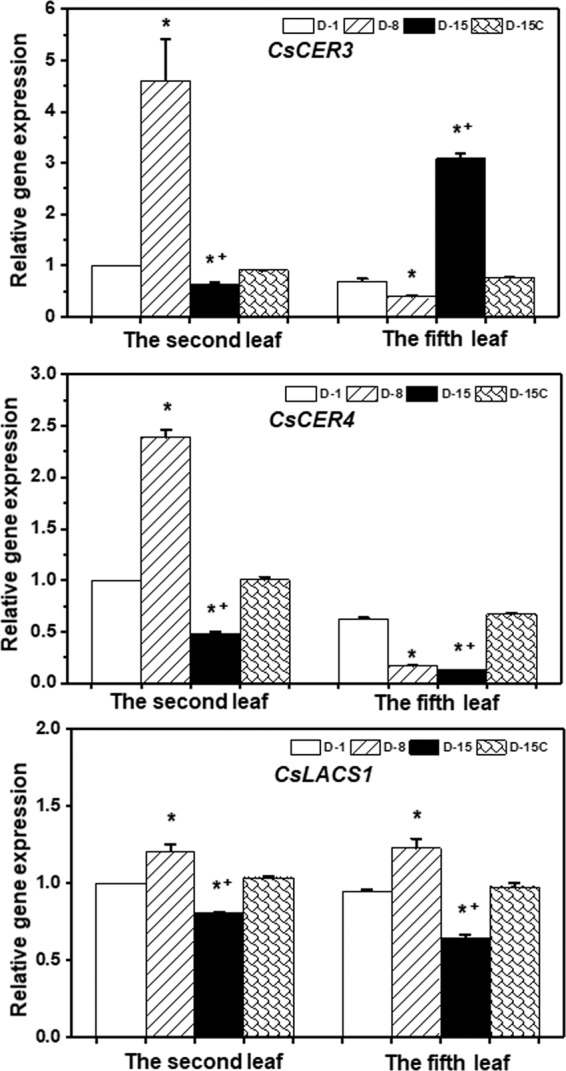
Expression levels of *CsCER3*, *CsCER4*, and *CsLACS1* during water deprivation treatment. D-1, D-8, and D-15 represent the first day, the eighth day, and the fifteenth day of water deprivation, respectively; D-15C: the well-watered control plants at day 15. *Indicates D-8 or D-15 was statistically significant compared with D-1 (p < 0.05); ^+^ indicates D-15 was statistically significant compared with D-8 (p < 0.05).

Kosma *et al*. (2009) reported that water deprivation activated *CER1* and *CER5* transcription in *Arabidopsis*^16^, which resulted in enhanced alkane biosynthesis. To uncover additional molecular regulation mechanisms underlying the drought-induced cuticle modification in tea tree, *CsCER3*, *CsCER4*, and *CsLACS1* were selected for transcriptional expression analysis. *CER3* and *CER4* are functioning in the alkane- and alcohol- formation pathway, respectively; while *LACS1* catalyzes the synthesis of ω-hydroxy fatty acyl-CoA intermediates in the pathway of cutin synthesis, lipid transmembrane transport, and intracellular trafficking^29–31^. In the tender leaf, *CsCER3* and *CsCER4* expression levels at D-8 were more than 3-, and 1-folds higher than that of D-1, while *CsLACS1* was slightly up-regulated; at D-15, *CsCER3*, *CsCER4*, and *CsLACS1* all were down-regulated. In the mature leaf, the expression of *CsCER3* and *CsCER4* at D-8 were reduced compared with that of D-1, while *CsLACS1* expression was slightly up-regulated. At D-15 *CsCER3* expression was 2-folds higher than that of D-1, while *CsCER4* and *CsLACS1* were down-regulated (Fig. 7). In *Arabidopsis* water deficit-treatment resulted in the down regulation of *CER4* and *LACS1*^16^. Our data demonstrated that the expression levels of *CsCER4* and *CsLACS1* were affected by leaf developmental stages as well as the severity of drought (Fig. 7). This may confer plants with greater flexibility for cuticle modification in different tissues or developmental stages in response to the severity of drought.

## Discussions

### The tender tea leaf was more responsive to water deficit than the mature leaf

In this study, four lines of evidence demonstrated that the tender tea leaf was hypersensitive to water deficit compared to the mature leaf: (1) Before the soil water content dropped to the lower threshold for optimum growth at D-8, wax crystals from the tender leaf were dramatically increased while the mature leaf was not affected (Fig. 2); (2) the adaxial cuticle thickness of the tender leaf increased at faster rate compared to that of the mature leaf (Fig. 3); (3) the wax coverage of the tender leaf increased at faster rate compared to that of the mature leaf during water deprivation treatment (Fig. 4a); (4) the expression levels of *CsCER3* and *CsCER4* of the tender leaf from D-1 to D-8 were highly upregulated while their expression levels from the mature leaf were down-regulated (Fig. 7). Since the tender leaf was hypersensitive to water deficient, this make its cuticle modification initiated earlier and quicker, thus make it more prepared for the upcoming water deficient (Fig. 6).

### Drought-induced cuticle structural modifications attribute to reduced leaf water loss

It’s widely assumed that cuticle water permeability is determined by the wax coverage or cuticle thickness. However, the experimental data only delivered equivocal results^9,16,23,32^. Nevertheless, majority of the drought stress treatments in different plant species revealed negative and significant correlations between epicuticular wax load and residual transpiration rates^16,19,23,33^. In this study, we also observed that cuticle thickness and wax coverage from both leaves had negative correlation with water loss rates (Figs. 3 and 6).

Drought stimulated the dramatic deposition of wax crystals on the adaxial surface of the tender leaf, but the mature leaf was only slightly affected (Fig. 2b). This deposition pattern may have eco-physiological relevance, since the tender leaves usually locate on the top of canopy thus receive direct solar irradiation. The wax crystals deposition on the adaxial surface of the tender leaf would help deflect incoming solar light, thus reduce leaf surface temperature. Higher temperature will increase water diffusion coefficient, thus enhance cuticle water permeance^34^. Consequently, the formation of dense wax crystal on the adaxial surface will reduce cuticular water permeance. Interestingly, the mature leaf formed wax flakes or lamellae at the abaxial surface (Fig. 3o). Riederer & Schneider (1990) suggested that on a microscopic scale the transport-limiting barrier within plant cuticles can be assumed to be made up of wax platelets of irregular outline and size, which may be arranged in one or several layers more or less parallel to the surface of the cuticle^32^. Under this hypothesis the lamellae crystals formed at the abaxial surface could improve cuticle barrier properties (Figs. 2n–o, 6). With the progression of water deprivation, electron-dense lamellate parallel to cuticular surface became increasingly visible (Fig. 3b,c,j,k). These structures were regarded to be cutin-rich^27^, suggesting that cutin biosynthesis could be induced by drought. The up-regulation of *CsLACS1* at D-8, a key gene for cutin biosynthesis, further support this notion (Fig. 7). In *Arabidopsis*, cutin monomer amount has positive correlation with cuticle barrier property^16^. However, it remains unknown if drought-induced cutin accumulation in tea tree contribute to the enhancement of cuticle barrier.

### The contributions of individual wax component for the enhancement of transpiration barrier under drought stress

Tea leaf cuticular waxes are remarkably complex with aliphatic VLCFA derivatives. To add this complexity, the tender leaf and the mature leaf showed marked difference in alicyclic compounds^3^. We found that drought induced dual wax compositional changes: 1) the wax coverage of each chemical class was altered by drought (Fig. 4b,c); 2) within each chemical class the chain length or the functional groups also were extensively modified (Figs. 5, S1 and S2). Wax composition is a critical factor influencing epidermal conductance rates through its effects on the water permeability coefficient^13^. The effects of hydrocarbon chains are the key factors to shape the transport barrier from long-chain aliphatic compounds^32^. For example, alkanes lack any polar substitution and are more hydrophobic substances, thus make alkanes play important roles for the transport barrier formation. Under normal growth condition tea leaf cuticle contained low level of alkanes (Fig. 4b,c). However, after water deprivation for 15 days alkanes became the dominant component in the tender leaf and the second most abundant component in the mature leaf (Fig. 4b,c). Similar increase in alkanes also were observed from *Arabidopsis*, cotton, alfalfa, and sesame under drought stress, and negatively correlated with leaf transpiration rate^16,20,22,35^. In this study we also found that drought treatment induced both shorter chain (C_17_ and C_19_) and the very long chain (C_37_ and C_39_) alkane biosynthesis, this led to significant expansion of alkane chain length distribution (Fig. 5); consequently, affected the weighted mean chain length and the root mean square deviation from the weighted average chain length, which are two parameters closely correlated to bulk and molecular physical properties of natural waxes^36,37^.

In this report, we found that the absolute glycol contents were up regulated with the progression of water deprivation (Fig. 4b,c), suggesting that glycols potentially are involved in the cuticle transpiration barrier improvement. At D-15, the appearance of longer chain length (C_24_ glycol) and the decrease of short chain length (C_16_ glycol) would also alter the weighted mean chain length and the deviation of chain length (Fig. S1b), thus could enhance the transpiration barrier (Fig. 6).

Triterpenoids were widely detected from intracuticular waxes of many different plant species, previous studies suggested that intracuticular triterpenoids did not contribute directly to the transpiration barrier^9,38,39^. Zhu *et al*.^3^ found that triterpenoids were absent from the tender tea leaf, and became dominant wax components with leaf maturation^3^. Here, we found that drought treatment did not affect triterpernoid levels of the tender leaf and the mature leaf (Fig. 4b,c). However, the leaf transpiration rates were significantly reduced from the drought-stressed leaves (Fig. 6). Seemingly, triterpenoids did not attribute to cuticle transpiration barrier, or the effects of triterpenoids were suppressed by other wax changes (Fig. 6). Under normal growth condition, β-amyrin and friedelin are the dominant triterpenoids, and were significantly reduced by drought treatment at D-15; meanwhile β-amyrone and betulin were detected at D-8, and canophyllol detected at D-15 (Fig. S2, Table S1). These observations raised possibility that individual triterpene may not be functionally equivalent for the formation of cuticle transpiration barrier. For example, β-amyrin has a small hydrophilic 3β-hydroxyl group, which was replaced by a ketone group in β-amyrone, thus make β-amyrone less polar than β-amyrin.

Although steroids widely present in cuticle of many different plant species, their contributions to cuticular transpiration barrier remains elusive. Analyses of ordered/disordered phases in reconstituted membranes demonstrated that plant sterols are able to form ordered phases in model membranes^40^, and maintain model membranes in a dynamic states less sensitive to abiotic stress^41^. Sterols have been reported to increase mechanical strength of the membrane^42^, to reduce passive membrane permeability of water and other small metabolites^43^. In this study, we found that the absolute steroid coverage as well as their composition were altered with the progression of water deprivation, the percentage of campesterol, stigmasterol and lanosterol were significantly decreased while β-sitosterol content was highly increased at D-15 (Figs. 4, S2). These data suggested that β-sitosterol could make specific contribution to the transpiration barrier. β-sitosterol is the direct precursor for stigmasterol synthesis, the dramatic accumulation of β-sitosterol with a concurrent slight increase of stigmasterol suggested that the hydrocarbon chain desaturation between C_22_-C_23_ was suppressed by drought stress (Table S1; Fig. S2), thus β-sitosterol possess a more saturated carbon chain compared to stigmasterol. Compared with campesterol side chain (CH_3_), β-sitosterol also has a longer side chain at C_24_(C_2_H_5_). In mammalian cell membranes, β-sitosterol and 24-methylcholesterol (campesterol) can regulate membrane fluidity and permeability in a similar manner to cholesterol; in contrast, stigmasterol might be specifically required for cell proliferation^44^. Based on these results, we suggest that sterols in plant cuticle might play similar structural roles as their counterparts of cell membrane. Riederer & Schneider (1990)^32^ suggested that the presence and amounts of non-aliphatic constituents (sterols and triterpenoids) may be important determinants for the susceptibility to mechanical stress of the wax barrier of plant cuticles. In this study, we observed that from D-8 to D-15 tea leaves started to lose turgor pressure temporally (Fig. 1), this would generate dramatic mechanical stress to leaf cuticle. The concurrent accumulation of β-sitosterol could enhance the flexibility and mechanical strength of cuticle, thus make cuticle withstand leaf turgor pressure changes and maintain cuticle integrity. These roles of sterols or triterpenoids can’t be easily resolved under normal growth conditions or when transpiration chamber method was used for transpiration measurement.

## Conclusions

In this study, we demonstrated that cuticles from the tender tea leaf and the fully expanded mature leaf were modified through several common and distinct modes in response to drought stress. The common mechanisms include increase in the wax coverage, cuticle thickness and osmiophilicity; adjustment of cuticular wax compositions, through the accumulation/reduction of same wax components, synthesis of new wax lipids, and expansion of the ranges of chain length distribution of alkanes and glycols. Results presented here also revealed that wax biosynthesis genes were differentially regulated depending on leaf developmental stages and the severity of drought stress, this may confer plants differential cuticular waxes remodeling even with same set of wax biosynthesis genes.

## Materials and Methods

### Plant materials

One-year old clonally propagated tea tree (*Camellia sinensis* cv *Jinmudan*) were purchased from Fuan Tea Breeding Base on March 2017, and transplanted into plastic pots with 25 cm in diameter and 25 cm in height, each pot was filled with same amount of red soil, three plants per pot. In total, 90 tea seedlings were transplanted into 30 pots. The pots were half-buried into tea garden and grown additional seven months before used for drought stress treatment.

### Water-deprivation treatment

To perform water-deprivation treatment tea pots were moved into a greenhouse. One day before the initiation of the water deprivation treatment all pots were fully irrigated with tap water to ensure all of them with same soil water content. The 30 pots were divided into control (5 pots, 15 plants) and drought treatment groups (25 pots, 75 plants). The control plants were irrigated daily with 500 mL of tap water per pot; the water was withheld from the drought treatment plants (Fig. S3). Each pot was weighed daily during the experiment; the data were used to calculate absolute soil water content. The air temperature and humidity inside the greenhouse were also recorded. The daily highest and lowest temperature and humidity were plotted (Fig. S4). During experiment, the highest and lowest temperature were in the ranges of 30–40 °C and 15–20 °C, respectively; the highest and lowest humidity were in the ranges of 67–75% and 37–45%, respectively. During night period the temperature gradually decreased to the lowest point, meanwhile the humidity was increased to the highest point.

### Leaf water loss measurement

To measure leaf water loss, the shoots with one bud and seven leaves were excised from 15-day drought treated plants and the well-watered control plants, lower part of the stems were immersed in tap water, and then kept in the dark overnight to equilibrate leaf water contents. Next day, leaf abaxial surfaces were evenly sprayed with 50 µM ABA, left in the dark for 1 h, and then excess water was gently blotted dry by soft tissue. The second leaf and the fifth leaf were then excised from stems, the initial water-saturated fresh weight (W_i_) was recorded, then leaves were kept in a controlled dark room (25 °C, 70% humidity), with weights determined hourly using a microbalance for a total of six hours (W_t1,2…6_). Lastly, the leaves were deactivated in 105 °C oven for 30 min followed by drying at 80 °C for 24 hours, then the dry weight of individual leaf was obtained (W_d_). Four biological replicates were used. Data were expressed as percentage of fully saturated leaf water content by using the formula: (W_i_ − W_t_) × 100/(W_i_ − W_d_).

### The scanning and transmission electron microscopy

The central part of the second and the fifth leaves, which were about 10- and 30-day-old after bud break, were harvested from well-watered control plants and drought treated plants at D-1, D-8, and D-15. The detailed protocols for sample preparation and electron microscopy observation followed the methods described by Zhu *et al*.^3^. Briefly, for SEM imaging, samples were air dried, small pieces of samples were fixed to sample holders, freeze dried, followed by sputter to coat a thin layer of gold, then observed under SEM (JEM-6380LV, JEOL, Japan). For TEM sample preparation, leaves were cut into small pieces, fixed in glutaraldehyde solution, rinsed with PBS buffer, post fixed with osmium tetroxide, then dehydrated through 30% and 50% ethanol, stained with uranyl acetate. Samples were further dehydrated with ethanol and acetone gradient, then infiltrated through a graded acetone/Epon/Spurr’s epoxy resin and polymerized. 70 nm thick sections were prepared and observed under transmission electron microscope (HT7700, Hitachi, Japan).

### Wax lipid analysis

The second leaf and the fifth leaf from well-watered control plants and the drought treated plants were harvested at D-1, D-8, and D-15. Cuticular waxes were extracted from whole leaves. The wax lipid isolation, derivatization, GC-MS, and GC-FID analysis followed the methods described by Racovita *et al*.^6^ and Zhu *et al*.^3^. Briefly, 10 individual leaves were randomly pooled together as one biological replicate, three biological replicates were used for each treatment. Leaves were photographed and leaf area calculated by Image J software. Leaves were extracted twice in chloroform, 30 s each with stirring. Extracts were combined and dried under nitrogen stream, then derivatized in 50 µL N, O-bis(trimethylsilyl)trifluoroacetamide (BSTFA, Aldrich, GC grade) plus 1% trimethylchlorosilane (Aldrich) (Aldrich, 99.8%, anhydrous). DB-1 (30 m × 0.25 mm × 0.25 μm, Agilent, California, USA) was used for wax analysis. GC-MS data were used for compound identification; FID data were used for quantification of individual wax homologs by normalize peak areas against that of the internal standard. Wax coverage was calculated based on the total area of the adaxial and abaxial surfaces.

### Gene expression analysis

The second leaf and the fifth leaf were harvested from the well-watered control and the drought treated plants at D-1, D-8, and D-15, frozen in liquid nitrogen immediately. Total RNA was isolated by using a modified CTAB method. 0.15 g of tea leaves were ground into power in the presence of liquid nitrogen and small amount of Polyvinylpyrrolidone (PVP), 0.9 mL of CTAB buffer and 45 µL of β-mercaptoethanol was added, incubated in 65 °C water bath for 30 min, centrifuged at 12000 g for 10 min at 4 °C, the supernatant was transferred into new tube, 1/3 volume of 5 M KAc was added, mixed well, incubated in ice-water bath for 10 min, then centrifuged at 12000 g for 20 min at 4 °C, the supernatant was transferred into new tube, equal volume of phenol:chloroform:isopropanol (25:24:1) was added, mixed well, incubated on ice-water bath for 10 min, then centrifuged at 12000 g for 20 min at 4 °C, the supernatant was transferred into new tube; above extraction step repeated once, the supernatant was transferred into new tube. Equal volume of chloroform:isopropanol (24:1) was added, mixed well, incubated on ice-water bath for 10 min, then centrifuged at 12000 g for 10 min at 4 °C, the supernatant was transferred into new tube. ½ volume of 8 M LiCl_2_ and 1% β-mercaptoethanol was added, mixed well, then stored in −20 °C freezer for 8 h. Centrifuged at 12000 g for 30 min at 4 °C, the supernatant was discarded. The pellet was rinsed twice with 1 mL of 75% ethanol, the dried pellet was dissolved in RNase-free water. CSA024836.1, CSA004251.1, and CSA028920.1 encode *Arabidopsis* homolog of *CER3*, *CER4*, and *LACS1* in *Camellia sinensis cv Yunkang10*^45^, their CDS were cloned from *Camellia sinensis cv Jinmudan*, and the sequences were deposited in GenBank with accession number of MH194573 (CsCER3), MH194572 (CsCER4), and MH194574 (CsLACS1). The primer sequences are: *CsCER3*_F: 5′-CGGCAGGGACACATTTCTATCA-3′, *CsCER3*_R: 5′-GCGTGAACAACACCTCTTTCG; *CsCER4*_F: 5′-AAGGGCGAGGAAGTATGGATG-3′, *CsCER4*_R: 5′-AATGATGGTGGGTCGGATGA; *CsLACS1*_F: 5′-GCATCTCCGCTCTGTGACAA-3′, *CsLACS1*_R: 5′-TCCACCACAGGTTTCAGTCAGA-3′; *CsGAPDH*_F: 5′-TTGGCATCGTTGAGGCTCT-3′, *CsGAPDH*_R: 5′-CAGTGGGAACACGGAAAGC-3′. Real time PCR was applied to quantify gene expression levels, *CsGAPDH* was used as internal control, and expression fold changes were calculated using 2^−ΔΔCt^ method. Three biological replicates were used for each treatment.

### Statistical analysis

Statistical analysis was performed by using Excel 2016 and Origin 2017, the data were expressed as mean ± standard error.

## Supplementary information

Supplementary information.

Table S1.
