# Supplementary material for: Drought stress modify cuticle of tender tea leaf and mature leaf for transpiration barrier enhancement through common and distinct modes
Source: Sci Rep. 2020 Apr 21;10:6696. doi: 10.1038/s41598-020-63683-4 (PMC7174317; doi:10.1038/s41598-020-63683-4)
Supplement: Supplementary file 1 — Supplementary information. [file 41598_2020_63683_MOESM1_ESM.pdf]

Drought stress modify cuticle of tender tea leaf and mature leaf for transpiration barrier enhancement through common and distinct modes

Mingjie Chen[1,3*#], Xiaofang Zhu[2,3#], Yi Zhang[3], Zhenghua Du[3], Xiaobing Chen[3], Xiangrui Kong[2], Weijiang Sun[4], Changsong Chen[2*]

[1]Henan Key Laboratory of Tea Plant Biology, College of Life Sciences, Xinyang Normal University, Xinyang 464000, China
[2]Tea Research Institute, Fujian Academy of Agricultural Sciences, Fuan, Fujian 355000, China
[3]Horticultural Plant Biology and Metabolomics Center, Haixia Institute of Science and Technology, Fujian Agriculture and Forestry University, Fuzhou, Fujian 350002, China
[4]Anxi College of Tea Science, Fujian Agriculture and Forestry University, Fuzhou, Fujian 350002, China
*Correspondence authors:
Email: mjchen@xynu.edu.cn； ccs6536597@163.com

Figure S1. Chain length distributions of esters and glycols during the course of drought treatment. D-1, D-8, and D-15 represent the first day, the eighth day, and the fifteenth day of water deprivation, respectively; D-15C: the well-watered control plants at day 15. * indicates D-8 or D-15 was statistically significant compared with D-1 ($p<0.05$); $^{+}$ indicates D-15 was statistically significant compared with D-8 ($p<0.05$).

Figure S2. Chain length distributions of terpenoids during the course of drought treatment. D-1, D-8, and D-15 represent the first day, the eighth day, and the fifteenth day of water deprivation, respectively; D-15C: the well-watered control plants at day 15. * indicates D-8 or D-15 was statistically significant compared with D-1 ($p<0.05$); $^{+}$ indicates D-15 was statistically significant compared with D-8 ($p<0.05$).

Figure S3 Diagram presentation of the drought treatment. D-1, D-8 and D-15 represent the first day, the eighth day and the fifteenth day of water deprivation, respectively; D-15C: the fifteenth day of well-watered control plants.

Figure S4 Greenhouse temperature and humidity changes during water-deprivation treatment. 1d, 8d and 15d represent the first day, the eighth day and the fifteenth day of water deprivation, respectively.

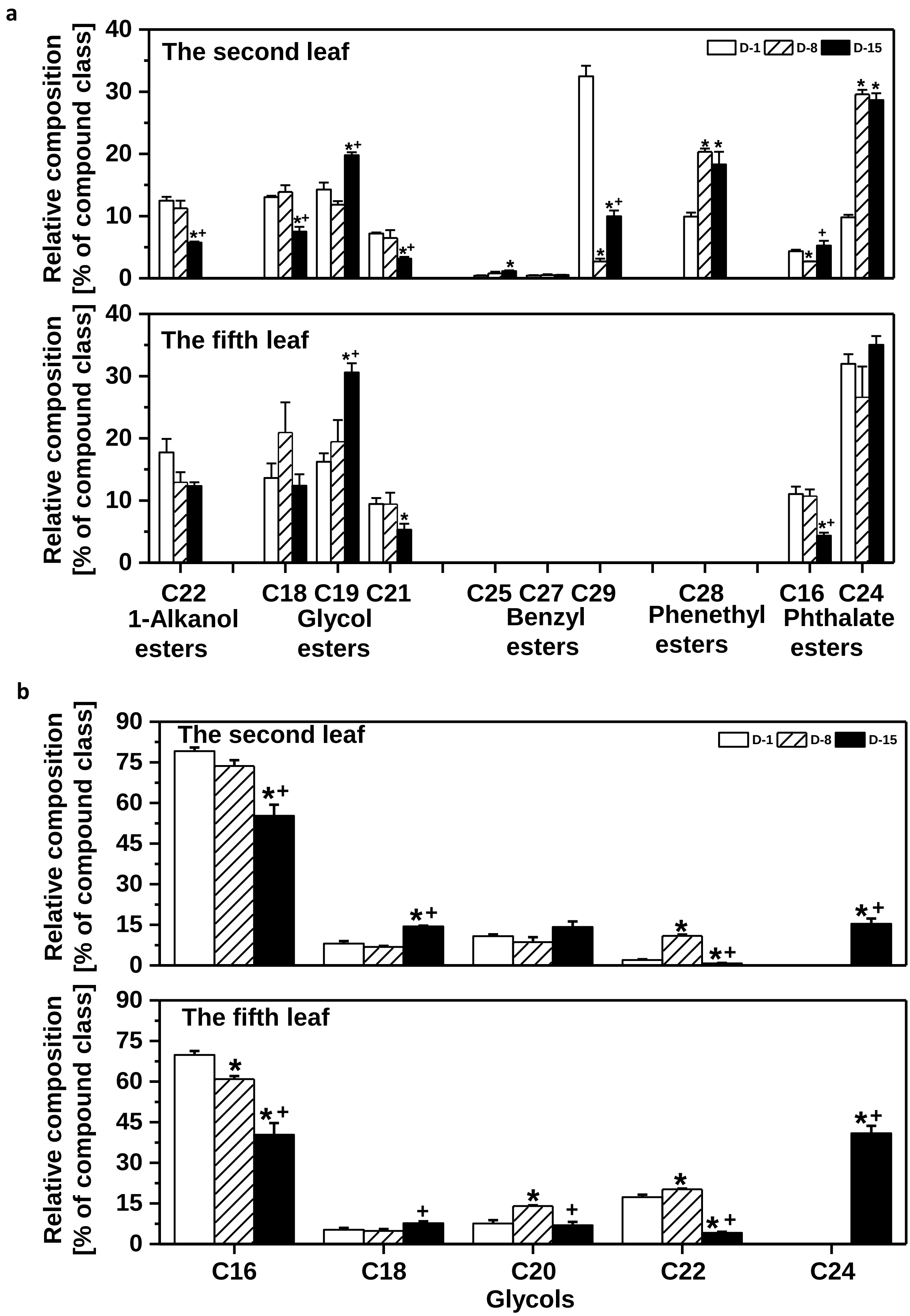

a
The second leaf
D-1 D-8 D-15
Relative composition [% of compound class]
The fifth leaf
C22
1-Alkanol esters
C18 C19 C21
Glycol esters
C25 C27 C29
Benzyl esters
C28
Phenethyl esters
C16 C24
Phthalate esters
b
The second leaf
The fifth leaf
C16 C18 C20 C22 C24
Glycols

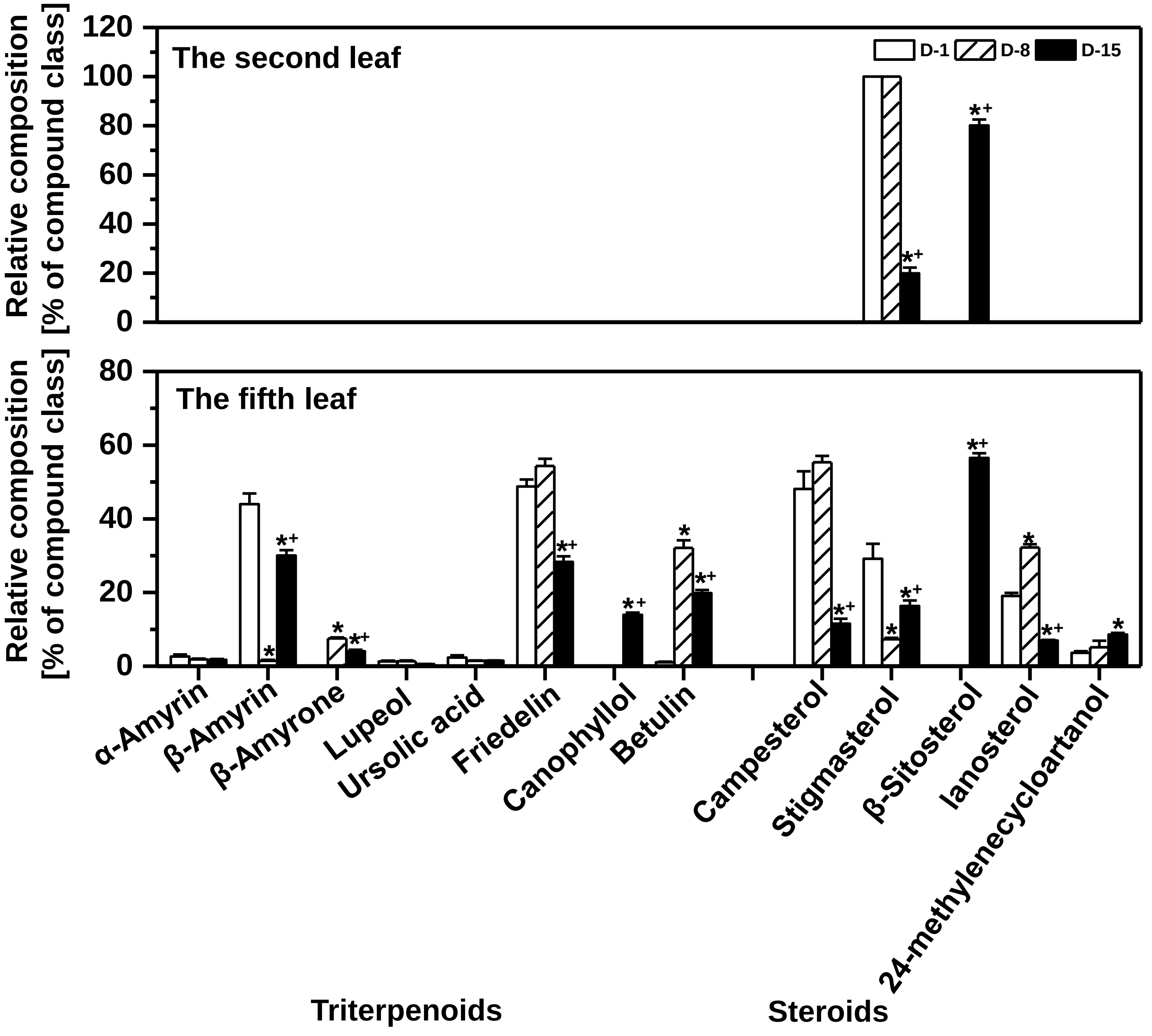

Relative composition [% of compound class]
The second leaf
D-1
D-8
D-15
The fifth leaf
α-Amyrin
β-Amyrin
β-Amyrone
Lupeol
Ursolic acid
Friedelin
Canophyllol
Betulin
Campesterol
Stigmasterol
β-Sitosterol
lanosterol
24-methylenecycloartanol
Triterpenoids
Steroids

Supplementary information

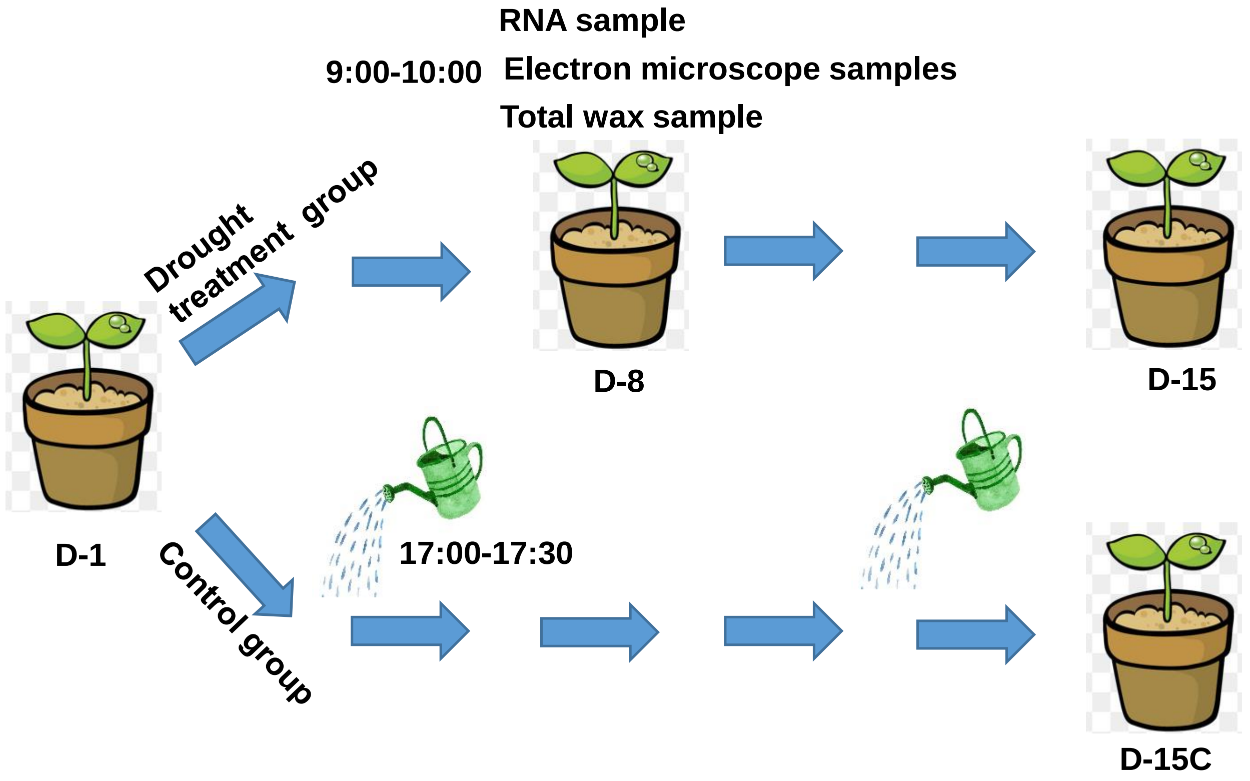

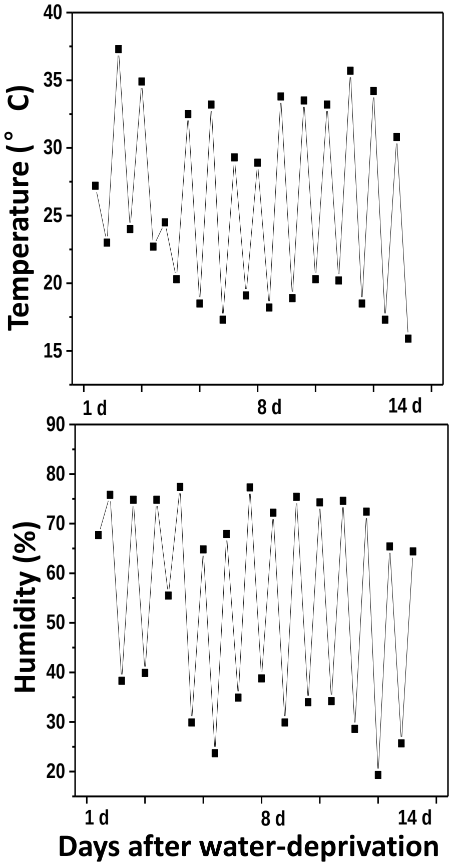
Temperature (° C)
40
35
30
25
20
15
1 d
8 d
14 d
Humidity (%)
90
80
70
60
50
40
30
20
1 d
8 d
14 d
Days after water-deprivation
