# Supplementary material for: Drought stress modify cuticle of tender tea leaf and mature leaf for transpiration barrier enhancement through common and distinct modes
Source: Sci Rep. 2020 Apr 21;10:6696. doi: 10.1038/s41598-020-63683-4 (PMC7174317; doi:10.1038/s41598-020-63683-4)
Supplement: Supplementary file 2 — Table S1. [file 41598_2020_63683_MOESM2_ESM.pdf]

Drought stress modify cuticle of tender tea leaf and mature leaf for transpiration barrier enhancement through common and distinct modes

Mingjie Chen[1,3*#], Xiaofang Zhu[2,3#], Yi Zhang[3], Zhenghua Du[3], Xiaobing Chen[3], Xiangrui Kong[2], Weijiang Sun[4], Changsong Chen[2*]
[1]Henan Key Laboratory of Tea Plant Biology, College of Life Sciences, Xinyang Normal University, Xinyang 464000, China
[2]Tea Research Institute, Fujian Academy of Agricultural Sciences, Fuan, Fujian 355000, China
[3]Horticultural Plant Biology and Metabolomics Center, Haixia Institute of Science and Technology, Fujian Agriculture and Forestry University, Fuzhou, Fujian 350002, China
[4]Anxi College of Tea Science, Fujian Agriculture and Forestry University, Fuzhou, Fujian 350002, China
***Correspondence authors:**
Email: mjchen@xynu.edu.cn; ccs6536597@163.com

			The second leaf								
			**D-1**		**D-8**		**D-15**				
			**Average(μg/cm^2)**	**SE**	**Average(μg/cm^2)**	**SE**	**Average(μg/cm^2)**	**SE**	**p（D-8 vs D1）**	**p（D-15 vs D-1）**	**p（D-15 vs D-8）**
**Acids**		C16	0.051703991	0.005414175	0.04492746	0.009867689	0.10689775	0.003240289	0.579591648	0.00094127	0.003962595
		C18	0.061709719	0.002238867	0.061434348	0.003578876	0.106808017	0.004672102	0.951120089	0.000959036	0.001523378
		C20	ND	ND	ND	ND	0.030792614	0.002876445	ND	0.000867376	0.000867376
		C22	0.006976454	0.001095768	0.011859825	0.002704231	0.022780266	0.002549337	0.169514069	0.001502834	0.008676464
		C24	0.009172235	0.001090299	0.007547967	0.002169889	0.018296413	0.001023001	0.540206192	0.003647896	0.010985772
		C26	0.027798663	0.003389037	0.016995613	0.006911889	0.034163704	0.002216189	0.233174979	0.191079276	0.077220237
		C28	0.067847125	0.007240988	0.019141843	0.001848627	0.040619389	0.004474323	0.002861653	0.032938277	0.011365287
		C30	0.031701972	0.00258749	0.023881455	0.003817574	0.043466944	0.004705608	0.165175382	0.093604919	0.03190781
	**Subtotal**		**0.25691016**	**0.01257994**	**0.185788511**	**0.025276409**	**0.403825098**	**0.005817128**	**0.065425176**	**0.000448308**	**0.001096032**
**1-Alkanols**		C18	0.023585024	0.002125514	0.020842528	0.003052777	0.0318212	0.002487808	0.501871794	0.065562565	0.049422511
		C24	0.008404569	0.000696101	0.04194388	0.005788097	0.023019833	0.001246343	0.004526598	0.000513065	0.033019694
		C26	0.028074537	0.003004404	0.017250353	0.006387787	0.036755004	0.000762405	0.199960008	0.048787433	0.038707972
		C28	0.321202831	0.019584329	0.237203553	0.045021707	0.196583303	0.011140538	0.162271603	0.00522122	0.430571011
		C30	0.440376551	0.007913174	0.771365329	0.023365458	0.309910872	0.0389395	0.000178482	0.030404538	0.000528175
		C32	0.067266921	0.006246782	0.078925891	0.014503543	0.078447017	0.006201784	0.501304258	0.272903026	0.977235373
		C34	0.014930515	0.002651763	0.029336085	0.006441681	0.039854914	0.002225008	0.107480509	0.001971823	0.197595144
	**Subtotal**		**0.903840947**	**0.025786829**	**1.196867619**	**0.086917952**	**0.716392144**	**0.050156293**	**0.03191314**	**0.029276096**	**0.008724728**
**Aldehydes**		C26	0.007765739	0.001344007	0.014855627	0.001319795	0.011398804	0.000395916	0.019708747	0.060492764	0.066144735
		C28	ND	ND	ND	ND	0.007873944	0.000718423	ND	0.000393709	0.000393709
		C30	0.002287467	0.000341664	0.006554824	0.001271392	0.00287526	0.000335622	0.03163079	0.287002351	0.04889796
	**Subtotal**		**0.010053206**	**0.001685052**	**0.02141045**	**0.002583537**	**0.022148008**	**0.000434873**	**0.021165782**	**0.002251808**	**0.792272868**
**Alkanes**		C17	ND	ND	ND	ND	0.031533189	0.003813761	ND	0.00116758	0.00116758
		C19	ND	ND	ND	ND	0.04305831	0.006483446	ND	0.002668072	0.002668072
		C21	0.026815015	0.002733052	0.037252107	0.003434383	0.117756757	0.007281468	0.076160148	0.000305894	0.000562084
		C25	0.026537965	0.001607373	0.036104004	0.002964082	0.177095275	0.003369396	0.047009175	2.25877E-06	6.11665E-06
		C27	0.014639211	0.002436426	0.014024746	0.001757209	0.13601354	0.004208138	0.847910618	1.52924E-05	1.16093E-05
		C29	0.069156382	0.002130479	0.110444635	0.023594284	0.285583705	0.014381391	0.156319178	0.000118579	0.003172599
		C31	0.009124373	0.000387448	0.009570985	0.000329188	0.107155579	0.003929085	0.429301898	1.56165E-05	1.58182E-05
		C35	0.023967889	0.002459016	0.025483204	0.006461339	0.091253993	0.005172895	0.837236963	0.000300367	0.001358257
		C37	ND	ND	ND	ND	0.053556071	0.004080438	ND	0.00019459	0.00019459
		C39	ND	ND	ND	ND	0.028649165	0.0037192	ND	0.00152833	0.00152833
	**Subtotal**		**0.170240835**	**0.010699596**	**0.232879681**	**0.027647431**	**1.071655584**	**0.034055965**	**0.102153428**	**1.46037E-05**	**4.40743E-05**

**1-Alkanol sesters**		C22	0.039275069	0.003912761	0.037649739	0.004023238	0.04911669	0.003429257	0.786506533	0.131509244	0.095903132
**Glycol esters**		C18	0.041100363	0.003456617	0.046475556	0.003693272	0.064372021	0.007873841	0.347856771	0.053744243	0.108726366
		C19	0.045258178	0.006435529	0.039626262	0.002420864	0.169641286	0.010466546	0.458727912	0.000535944	0.00026739
		C21	0.022696697	0.002050502	0.021869055	0.004938228	0.026878296	0.001726454	0.884486715	0.193775912	0.392515771
**Benzyl esters**		C25	0.001261049	0.000257982	0.00258818	0.000821924	0.0096924	0.000943184	0.198271376	0.000994589	0.004746279
		C27	0.001314746	0.000119964	0.001690494	0.000570746	0.004456757	0.000440086	0.554487471	0.002328329	0.018487498
		C29	0.101576577	0.005616739	0.00894554	0.001301948	0.084859089	0.00535642	0.097550318	0.055131582	0.024734824
**Phenethyl esters**		C28	0.031057817	0.002055956	0.068169413	0.0025868	0.156678304	0.019764788	0.000357954	0.003203614	0.011331834
**Phthalate esters**		C16	0.005482511	0.000605888	0.005588101	0.000569302	0.022613482	0.003681443	0.905065501	0.010094924	0.010258273
		C16	0.008087674	0.000763505	0.00347724	9.74955E-05	0.02254948	0.003886389	0.00390665	0.02174559	0.008009967
		C24	0.026837817	0.003473914	0.062906908	0.010048772	0.210456039	0.00807682	0.027468957	3.10651E-05	0.000332618
		C24	0.00410975	0.000276341	0.036587298	0.006032929	0.034497002	0.003097328	0.005777345	0.000614459	0.7732904
	**Subtotal**		**0.328058247**	**0.022994542**	**0.335573786**	**0.015089326**	**0.855810844**	**0.033209285**	**0.798182968**	**0.000198101**	**0.000140379**
**Glycols**		C16	0.179751083	0.009082939	0.248098011	0.034447684	0.195140427	0.029565519	0.12749022	0.644900041	0.30818798
		C18	0.018383711	0.002334608	0.022543593	0.001280216	0.05031979	0.003343769	0.193238453	0.001435736	0.001487916
		C20	0.024482064	0.001802156	0.027958083	0.004172073	0.048478869	0.003808008	0.486988894	0.004693685	0.022103587
		C22	0.004550542	0.00039788	0.036869682	0.005615878	0.002647006	0.000611649	0.004562585	0.059498014	0.003747719
		C24	ND	ND	ND	ND	0.05258835	0.003424541	ND	0.000104912	0.000104912
	**Subtotal**		**0.227167399**	**0.010792683**	**0.335469369**	**0.03955766**	**0.349174442**	**0.026575189**	**0.057503156**	**0.013120192**	**0.7879478**
**Tocopherols**		γ-Tocopherole	0.004542747	0.000718784	0.027144864	0.001805387	0.004595012	0.000241457	0.000312271	0.948355233	0.00024468
		β-Tocopherole	ND	ND	ND	ND	ND	ND	ND	ND	ND
	**Subtotal**		**0.004542747**	**0.000718784**	**0.027144864**	**0.001805387**	**0.004595012**	**0.000241457**	**0.000312271**	**0.948355233**	**0.00024468**
**Triterpenoids**		α-Amyrin	ND	ND	ND	ND	ND	ND	ND	ND	ND
		β-Amyrin	ND	ND	ND	ND	ND	ND	ND	ND	ND
		β-Amyrone	ND	ND	ND	ND	ND	ND	ND	ND	ND
		Lupeol	ND	ND	ND	ND	ND	ND	ND	ND	ND
		Ursolic acid	ND	ND	ND	ND	ND	ND	ND	ND	ND
		Friedelin	ND	ND	ND	ND	ND	ND	ND	ND	ND
		Canophyllol	ND	ND	ND	ND	ND	ND	ND	ND	ND
		Betulin	ND	ND	ND	ND	ND	ND	ND	ND	ND
	**Subtotal**		**ND**	**ND**	**ND**	**ND**	**ND**	**ND**	**ND**	**ND**	**ND**
**Steroids**		Campesterol	ND	ND	ND	ND	ND	ND	ND	ND	ND
		Stigmasterol	0.012977029	0.001070845	0.003940869	0.00040873	0.043400905	0.004219906	0.001399743	0.002206124	0.000741546
		β-Sitosterol	ND	ND	ND	ND	0.176109531	0.009907689	ND	5.88573E-05	5.88573E-05
		Lanosterol	ND	ND	ND	ND	ND	ND	ND	ND	ND

		24-methylenecycloartanol	ND	ND	ND	ND	ND	ND	ND	ND	ND
	**Subtotal**		**0.012977029**	**0.001070845**	**0.003940869**	**0.00040873**	**0.219510436**	**0.007149749**	**0.001399743**	**8.93476E-06**	**7.25454E-06**
**Unidentified**			0.096697882	0.007120985	0.395693583	0.017589738	0.24812657	0.003727087	9.47942E-05	4.67375E-05	0.001201081
	**Subtotal**		**0.096697882**	**0.007120985**	**0.395693583**	**0.017589738**	**0.24812657**	**0.003727087**	**9.47942E-05**	**4.67375E-05**	**0.001201081**
	**Total**		**2.010488452**	**0.086620485**	**2.734768732**	**0.15312798**	**3.891238138**	**0.054481803**	**0.014649765**	**5.15599E-05**	**0.002061747**

			**The fifth leaf**								
			**D-1**		**D-8**		**D-15**				
			**Average(μg/cm)**	**SE**	**Average(μg/cm)**	**SE**	**Average(μg/cm)**	**SE**	**p（D-8 vs D-1）**	**p（D-15 vs D-1）**	**p（D-15 vs D-8）**
**Acids**		C16	0.053614986	0.005903244	0.034804899	0.007301476	0.052626871	0.007666428	0.115674171	0.923574728	0.167590134
		C18	0.055048568	0.006265918	0.045836558	0.013001978	0.062810518	0.010210504	0.558016192	0.552350443	0.36257548
		C20	ND	ND	ND	ND	0.037373369	0.001958706	ND	4.44497E-05	4.44497E-05
		C22	ND	ND	ND	ND	0.008398127	0.001690149	ND	0.007657647	0.007657647
		C24	0.00611376	0.000362788	0.007017256	0.00149343	0.004541195	0.000963106	0.588197988	0.201234581	0.235947012
		C26	0.002985804	0.000198467	0.003146595	0.000259444	0.012468213	0.000852779	0.648342738	0.000412428	0.000472498
		C28	0.006463793	0.000821515	0.016386334	0.001784802	0.013111772	0.001643446	0.007230199	0.022391533	0.24844824
		C30	ND	ND	0.03910054	0.003399741	0.025242404	0.001208868	0.000326307	3.10837E-05	0.018449122
	**Subtotal**		**0.124226912**	**0.01213331**	**0.146292182**	**0.025698354**	**0.216572468**	**0.020462812**	**0.480846103**	**0.017814563**	**0.099152312**
**1-Alkanols**		C18	0.020257389	0.00465226	0.025170349	0.007599221	0.022523496	0.003036005	0.610720672	0.704221404	0.762560125
		C24	0.014351331	0.001648277	0.02020756	0.003139698	0.056089263	0.03448359	0.173985691	0.293240787	0.358615324
		C26	0.005998025	0.001215098	0.002139732	0.000637824	0.015117326	0.002609803	0.048243443	0.033931345	0.008458413
		C28	0.021206787	0.005033873	0.043664496	0.003867601	0.036073435	0.006216777	0.024062884	0.136637149	0.358392894
		C30	ND	ND	ND	ND	ND	ND	ND	ND	ND
		C32	0.081534841	0.013338789	0.105915028	0.008198972	0.080522867	0.003575271	0.194432274	0.945101202	0.046923268
		C34	ND	ND	ND	ND	ND	ND	ND	ND	ND
	**Subtotal**		**0.143348373**	**0.021450246**	**0.197097166**	**0.004814927**	**0.210326388**	**0.028929166**	**0.070832989**	**0.136435817**	**0.675296903**
**Aldehydes**		C26	0.00631801	0.000223522	0.008761163	0.001894388	0.007423927	0.001438528	0.269485766	0.489761644	0.604004585
		C28	0.004497763	0.000305692	0.004995349	0.000102331	0.003681158	0.000763565	0.197572625	0.3769808	0.163227764
		C30	0.015232574	0.002457038	0.01798273	0.001401328	0.008603024	0.000943993	0.385966782	0.065447901	0.00515227
	**Subtotal**		**0.026048346**	**0.002254222**	**0.031739242**	**0.002916473**	**0.01970811**	**0.003094641**	**0.197495777**	**0.173061036**	**0.047379672**
**Alkanes**		C17	ND	ND	ND	ND	0.018621144	0.003301551	ND	0.004864667	0.004864667
		C19	ND	ND	ND	ND	0.034228107	0.003925297	ND	0.000952709	0.000952709

		C21	0.03155043	0.001312267	0.03327211	0.005287769	0.087835045	0.003394007	0.767796894	0.000101965	0.000967962
		C25	0.029973189	0.001271147	0.031668191	0.002317966	0.125660718	0.006890988	0.556307296	0.000166552	0.000206482
		C27	0.018780368	0.000883492	0.017912453	0.000560907	0.104071165	0.004827944	0.453533165	6.43686E-05	5.94964E-05
		C29	0.037598229	0.003274145	0.055597952	0.009384055	0.172599954	0.011082535	0.14437111	0.000306981	0.001288624
		C31	0.012349861	0.000695295	0.019404176	0.008252323	0.074327291	0.007046581	0.442314051	0.000939009	0.007173813
		C35	0.02099458	0.00281011	0.02459069	0.001832231	0.073338075	0.004756282	0.344101928	0.000692242	0.000667717
		C37	ND	ND	ND	ND	0.04643036	0.004579479	ND	0.000532787	0.000532787
		C39	ND	ND	ND	ND	0.024072547	0.001808213	ND	0.000184035	0.000184035
	**Subtotal**		**0.151246656**	**0.009711451**	**0.182445572**	**0.01901734**	**0.761184405**	**0.047718736**	**0.217794171**	**0.000233766**	**0.000353623**
**1-Alkanol sesters**		C22	0.038907483	0.001971861	0.026303277	0.002382392	0.054260514	0.006663471	0.015152652	0.091692135	0.016808358
**Glycol esters**		C18	0.031460622	0.008440655	0.043084799	0.010966991	0.055296874	0.012725595	0.448206672	0.193558898	0.507523837
		C19	0.036853964	0.006348706	0.040076405	0.007848012	0.132834752	0.005070123	0.76552841	0.000293897	0.000577994
		C21	0.021539286	0.004252685	0.019380037	0.004310131	0.023140535	0.004134502	0.73940088	0.800539739	0.563109305
**Benzyl esters**		C25	ND	ND	ND	ND	ND	ND	ND	ND	ND
		C27	ND	ND	ND	ND	ND	ND	ND	ND	ND
		C29	ND	ND	ND	ND	ND	ND	ND	ND	ND
**Phenethyl esters**		C28	ND	ND	ND	ND	ND	ND	ND	ND	ND
**Phthalate esters**		C16	0.005079203	0.000676348	0.007636508	0.001165965	0.008794845	0.001220949	0.130659368	0.056268178	0.530342016
		C16	0.019143939	0.000235022	0.014710678	0.00283488	0.010536967	0.002450973	0.194122309	0.024995126	0.327803746
		C24	0.038829276	0.002285654	0.028465393	0.012863705	0.126192716	0.000812423	0.472033544	3.54801E-06	0.00162275
		C24	0.032201418	0.001591422	0.026404948	0.003054389	0.026059841	0.006094457	0.167662181	0.384752233	0.96205205
	**Subtotal**		**0.224015192**	**0.020480234**	**0.206062044**	**0.015899074**	**0.437117045**	**0.033704248**	**0.526757682**	**0.005679489**	**0.003441469**
**Glycols**		C16	0.182569197	0.013922075	0.119741781	0.014378932	0.136355455	0.023267687	0.034877816	0.163514239	0.576378431
		C18	0.013740964	0.00218023	0.009283678	0.000837133	0.025422905	0.001480682	0.128960118	0.011399515	0.000688473
		C20	0.019658442	0.003104398	0.027445003	0.002934383	0.022625559	0.002863742	0.142412943	0.521086485	0.305015507
		C22	0.045140195	0.003602856	0.039449694	0.003708329	0.013814789	0.001847284	0.332848313	0.001503139	0.00346731
		C24	ND	ND	ND	ND	0.135317083	0.004072946	ND	4.89505E-06	4.89505E-06
	**Subtotal**		**0.261108798**	**0.017151755**	**0.195920155**	**0.021092151**	**0.333535792**	**0.023185461**	**0.074525646**	**0.065963404**	**0.011778041**
**Tocopherols**		γ-Tocopherole	ND	ND	ND	ND	ND	ND	ND	ND	ND
		β-Tocopherole	0.009629117	0.002680511	0.004941127	0.000559712	0.009458363	0.000987901	0.162062109	0.955204273	0.016423089
	**Subtotal**		**0.009629117**	**0.002680511**	**0.004941127**	**0.000559712**	**0.009458363**	**0.000987901**	**0.162062109**	**0.955204273**	**0.016423089**
**Triterpenoids**		α-Amyrin	0.029597907	0.005789446	0.02547402	0.001458906	0.023014925	0.002533893	0.527733787	0.356383846	0.447664061
		β-Amyrin	0.511438057	0.054919778	0.01936505	0.002178569	0.399877727	0.033432106	0.00086105	0.157734626	0.000342682
		β-Amyrone	ND	ND	0.106404879	0.015531943	0.053519213	0.004976587	0.002376318	0.000423848	0.031596653
		Lupeol	0.01468671	0.002585584	0.019330554	0.005959334	0.007583102	0.000755502	0.514194526	0.057754213	0.122167333
		Ursolic acid	0.026602901	0.006638694	0.019919969	0.000465815	0.019885171	0.000428166	0.372103859	0.369711053	0.958776245
		Friedelin	0.562646918	0.005029885	0.765187392	0.085090296	0.375529224	0.018595943	0.076308026	0.000628946	0.011042701

		Canophyllol	ND	ND	ND	ND	0.184686899	0.011010266	ND	7.40249E-05	7.40249E-05
		Betulin	0.012266315	0.001292928	0.451139512	0.052978645	0.263175905	0.012168485	0.001160477	3.34136E-05	0.02586789
	**Subtotal**		**1.157238807**	**0.045563695**	**1.406821376**	**0.146944758**	**1.327272165**	**0.049053182**	**0.180062966**	**0.064000128**	**0.634664326**
**Steroids**		Campesterol	0.035082644	0.004928747	0.036705122	0.005228402	0.022606288	0.002722012	0.832421573	0.091028979	0.075016997
		Stigmasterol	0.020902733	0.002188985	0.004766319	0.000275274	0.032705376	0.005703167	0.001858873	0.125517181	0.008083586
		β-Sitosterol	ND	ND	ND	ND	0.110983255	0.007848096	ND	0.000145157	0.000145157
		Lanosterol	0.013714633	0.000338978	0.021152077	0.002068654	0.01366026	0.000954594	0.023841649	0.959767509	0.03026211
		24-methylenecycloartanol	0.002605286	0.000191201	0.003311837	0.000992633	0.017005405	0.002021744	0.523092579	0.002088486	0.003698659
	**Subtotal**		**0.072305297**	**0.002960096**	**0.065935355**	**0.007603998**	**0.196960584**	**0.017665213**	**0.487410404**	**0.00227862**	**0.00242613**
**Unidentified**			1.34469464	0.034405859	1.444171487	0.121401102	0.847788427	0.02411144	0.474582326	0.00029254	0.008533027
	**Subtotal**		**1.34469464**	**0.034405859**	**1.444171487**	**0.121401102**	**0.847788427**	**0.02411144**	**0.474582326**	**0.00029254**	**0.008533027**
	**Total**		**3.513862139**	**0.082507194**	**3.881425707**	**0.281313686**	**4.359923746**	**0.075575075**	**0.278197937**	**0.001639351**	**0.175790838**
